# Exposure of Chlorpromazine to 266 nm Laser Beam Generates New Species with Antibacterial Properties: Contributions to Development of a New Process for Drug Discovery

**DOI:** 10.1371/journal.pone.0055767

**Published:** 2013-02-06

**Authors:** Mihail Lucian Pascu, Balazs Danko, Ana Martins, Nikoletta Jedlinszki, Tatiana Alexandru, Viorel Nastasa, Mihai Boni, Andra Militaru, Ionut Relu Andrei, Angela Staicu, Attila Hunyadi, Seamus Fanning, Leonard Amaral

**Affiliations:** 1 Laser Department, National Institute for Laser, Plasma and Radiation Physics, Magurele/Ilfov, Romania; 2 Institute of Pharmacognosy, Faculty of Pharmacy, University of Szeged, Szeged, Hungary; 3 Centres for Food Safety & Food-borne Zoonomics, School of Agriculture, Food Science & Veterinary Medicine, University College Dublin, Dublin, Ireland; 4 Group of Mycobacteria, Unit of Microbiology, Institute of Hygiene and Tropical Medicine, Universidade Nova de Lisboa, Lisbon, Portugal; 5 Faculty of Physics, University of Bucharest, Magurele/Ilfov, Romania; UMR-S665, INSERM, Université Paris Diderot, INTS, France

## Abstract

**Introduction:**

Phenothiazines when exposed to white light or to UV radiation undergo a variety of reactions that result in degradation of parental compound and formation of new species. This process is slow and may be sped up with exposure to high energy light such as that produced by a laser.

**Methods:**

Varying concentrations of Chlorpromazine Hydrochloride (CPZ) (2–20 mg/mL in distilled water) were exposed to 266 nm laser beam (time intervals: 1–24 hrs). At distinct intervals the irradiation products were evaluated by spectrophotometry between 200–1500 nm, Thin Layer Chromatography, High Pressure Liquid Chromatography (HPLC) - Diode Array Detection, HPLC tandem mass spectrometry, and for activity against the CPZ sensitive test organism *Staphylococcus aureus* ATCC 25923.

**Results:**

CPZ exposure to 266 nm laser beam of given energy levels yielded species, whose number increased with duration of exposure. Although the major species produced were Promazine (PZ), hydroxypromazine or PZ sulfoxide, and CPZ sulfoxide, over 200 compounds were generated with exposure of 20 mg/mL of CPZ for 24 hrs. Evaluation of the irradiation products indicated that the bioactivity against the test organism increased despite the total disappearance of CPZ, that is due, most probably, to one or more new species that remain yet unidentified.

**Conclusions:**

Exposure of CPZ to a high energy (6.5 mJ) 266 nm laser beam yields rapidly a large number of new and stable species. For biological grade phenothiazines (in other words knowing the impurities in the samples: solvent and solute) this process may be reproducible because one can control within reasonably low experimental errors: the concentration of the parent compound, the laser beam wavelength and average energy, as well as the duration of the exposure time. Because the process is “clean” and rapid, it may offer advantages over the pyrogenically based methods for the production of derivatives.

## Introduction

A very large number of medicinal compounds developed for various indications during the twentieth century have their origins in the heterocyclic group phenothiazine. The dye methylene blue, the first phenothiazine, was created in middle nineteenth century and studied extensively by Ehrlich [1]. Among the many properties defined for methylene blue was the ability to prevent the mobility of certain bacteria. This observation led to the discovery that when humans are given the dye, they become lethargic [2]. The dye was also shown by Ehrlich to cure malaria and as of today, this property is being exploited for therapy of drug resistant malaria [3]. Among the many phenothiazines that have reached the pharmaceutical market place, is Chlorpromazine (CPZ), the first neuroleptic drug which was discovered by accident when a French surgeon used another phenothiazine, promethazine, together with pepthidine as part of a lytic cocktail to induce relaxation and indifference in surgical patients. This mixture perked some interest among chemists of the pharmaceutical company Rhone Poulenc and in 1953 the phenothiazine Lactargil (chlorpromazine) was released for use as a neuroleptic. During the 1950's and continued to this date, the global use of CPZ has generated huge numbers of studies that resulted in over 20,000 research articles (PubMed), and among the major medical discoveries made was the observation that it could cure the aortus monkey of malaria [3] and humans infected with tuberculosis [4], [5]. Derivatives of CPZ include thioridazine (THR), the neuroleptic that replaced CPZ for therapy of psychosis since it produced fewer and less serious side effects than its parental compound. THR is now being used for the therapy of extensively drug resistant tuberculosis as recommended from the studies performed by Amaral and his group [6]–[14].

On the other hand, it was shown [15] that in rats after ingestion in the organism, the CPZ is mainly converted to the CPZ sulfoxide, by natural processes related to metabolism and a part only of the parent compound may reach the aquatic environment. Looking at CPZ sulfoxide as a metabolite, this demonstrates that by natural processes one may obtain metabolites which may be produced in laboratory as well. For instance, the biotransformation of β-adrenoceptor antagonist propranolol metabolized in vitro with S9 rat liver fraction is reported in [16] by Nalec -Jawecky, but as in [15], no interaction of the compound with laser light was reported.

Studies about the interaction of several classes of medicines with incoherent or coherent optical beams followed by the generation of reaction products are reported in the last decades. It was shown that the primary species produced by flash photolysis at 266 nm of AMI-HCl (amitriptyline) and NT-HCl (nortriptyline) solutions are the solvated electron formed *via* a biphotonic photoionization and the corresponding radical cation; the studied solutions were nitrogen saturated to avoid the production of singlet oxygen [17]. The protriptyline hydrochloride (PTL-HCl) showed (by 266 nm nanosecond laser flash photolysis) the presence of the triplet−triplet transient intermediate at low laser beam intensities and two transient intermediates (the solvated electron and the radical cation) at high laser beam energy. The authors considered that the PTL-HCl triplet state may be associated with the in vivo phototoxic effects of this drug [18]. In line with these experiments, the tricyclic antidepressants (TCAs) - Imipramine (IPA), desimipramine (DIPA) and clomipramine (CIPA) - photophysical properties were studied in different solvents [19]. It was shown that the primary transient intermediates produced by 266 nm high-intensity laser photolysis are the solvated electron and the corresponding radical cation, consistent with the results reported in [18]. Results reported in [20] show that exposure to UV of protriptyline hydrochloride (PTL) 10^−3^ M solutions in phosphate-buffered saline, resulted in a mixture of at least five product which caused red blood cell lysis but required water for their formation and were stable for at least one week.

In the case of Chlorprothixene (CPTX), in vitro irradiation induces a Z/E isomerization, which can affect its pharmacokinetic properties. This photoisomerization is not dependent on the oxygen concentration in the solutions. In the presence of water, 2-chlorothioxanthone (CTX) is produced after prolonged irradiation and it seems that the phototoxicity of zCPTX is somehow affected by the formation of CTX [21].

Particular studies on the photophysical properties of phenothiazines and their derivatives were reported, related mainly to their phototoxic effects while administrated as antipsychotic drugs.

In [22] are shown absorption, steady-state, and time-resolved emission, laser flash photolysis, and quantum theoretical results for the ground state, the first excited singlet and triplet states, and the cation radical in the case of: promazine hydrochloride, 2-chlorpromazine hydrochloride, 2-trifuoromethylpromazine hydrochloride, 2-trifluoromethylperazine dihydrochloride, 2-thiomethylpromazine), and thioridazine hydrochloride. The most important effect was evidenced for the triplet state of the 2-halogenated derivatives in phosphate buffer, not in water only. This triplet state is quenched by a proton-transfer mechanism. In [23], in order to understand the mechanisms that trigger the phototoxic response to 2-chlorophenothiazine derivatives, the relationship between the molecular structure of halogenated phenothiazines and their phototoxic activity, their photophysics and photochemistry were studied in several alcohols and anaerobic conditions, under exposure at 313 nm optical radiation. The conclusions were that the photodestruction quantum yields of the studied derivatives are the same under the same conditions of solvent and irradiation wavelength and that the quantum yield depends on the utilized solvent.

Starting from the fact that CPZ is known to self-associate, the self-association and cyclodextrin binding properties of CPZ have been studied via fluorescence and UV-VIS absorption measurements and the effect on pharmacokinetic parameters in rats when dosed with a SBE(7)-beta-CD containing formulation was evaluated [24]. It was found that the SBE(7)-beta-CD binding constant of CPZ is strongly depending on concentration and the variation can be attributed to the self-association of CPZ.

Attempts to understand the interaction mechanisms between the phenothiazines and the biological targets were made, with some significant success. In [25] one reports the red blood cell lysis photosensitized by CPZ and protriptyline. It was observed that when the two compounds were irradiated in the absence of red cells, both formed photoproducts which lysed the cells indicating that a process of photosensitizing of the membrane disruption by mechanisms that are independent of oxygen is produced.

The photoaddition of CPZ to DNA as a possible mechanism to explain the CPZ photobiologic effects was also studied. The obtained results indicate that formation of a complex between CPZ and the double-stranded DNA absorbing at 340 nm is protecting CPZ from photodecomposition and inhibits the covalent photoadduct formation [26]. In a further study [27], it was investigated if CPZ molecules that are intercalated between base pairs in double-stranded DNA are the absorbing species for the photoaddition reaction. The resulting data indicated that the absorption spectrum of the CPZ-DNA complex is correlated closely with the absorption spectrum of nonintercalated CPZ rather than with the spectrum of intercalated CPZ and this suggested that the latter species is not the chromophore for the photoaddition reaction.

Studies on the photochemistry of the phenothiazine family have produced a series of reports with different and, most of the time, contradictory results. This fact is mainly due to the differences between the experimental conditions used for studies and the drug concentrations used in them.

On the other hand, CPZ since its creation is known to undergo photolysis when exposed to white light [28] and especially UV [29]. A detailed analysis of the results reported in [29] shows that, in a broad sense, effects of the same kind as those reported in this paper were evidenced, but significant differences between data in [29] and our data may be pointed out as presented in the section Discussion.

Interest in the reaction(s) of CPZ under white light and UV resulted from the observation that some patients who were treated with the compound develop dermal and ocular photosensitivity [30], [31]. Consequently, the effect of light on CPZ has been studied extensively. The effect of laser beams on the structure of CPZ has been previously studied [32], [33]. These studies suggested that using laser flash photolysis, the photoionization of CPZ during S_1_ excitation (wavelength greater than 300 nm) is a stepwise biphotonic process. Significant data are reported in a paper showing that after CPZ exposure to light the formation of stable, toxic photoproducts takes place, which causes cell membrane disruption. These toxic photoproducts were characterized as dimers and higher multimers of CPZ. The exposure of CPZ solutions to radiation emitted by a medium pressure Hg lamp filtered to exclude λ<280 nm has shown that the obtained CPZ photoproducts which cause cell membrane disruption are dimers and higher multimers [34].

In a reference paper, the interaction of some phenothiazines with laser beams, the mechanism for photoionization of promazine (PZ) and CPZ has been studied as a function of solvent and excitation conditions [35]. It was shown that sequential biphotonic absorption is responsible for photoionization induced by irradiation with pulsed laser beams at 308 or 355 nm and that photoionization is solvent dependent upon excitation of the triplet state at 355 nm or near its absorption maximum of 460 nm. It also resulted that during single wavelength pulsed irradiation, the second photon is absorbed predominantly by the lowest excited singlet state rather than the lowest excited triplet state. Based on this first conclusion, picosecond excitation was used at two distinct wavelengths and it was demonstrated that excitation of both lowest singlet and triplet states can lead to photoionization in aqueous solution. Although CPZ and PZ have similar structure, differing only in the chlorine substituent at the **2** position in CPZ, the triplet states of these compounds show markedly different behavior in aqueous solution. More, studies in methanol and deaerated aqueous solutions have shown that the CPZ triplet state has a very short lifetime in water.

A study of interest for our experiments is reported in [36] where the micellar properties of promazine hydrochloride, at different concentrations of NaCl and cationic surfactants are presented. It is shown that because the drug molecules contain a hydrophobic ring structure with a hydrocarbon chain and an amine group, they behave like surfactants and self-associate in water above the critical micelle concentration (*cmc*). It is also shown that the *cmc* of amphiphiles (drugs or surfactants) depends on their molecular structure and on the physicochemical conditions they are kept in such as: pH, temperature, ionic strength, additive concentration.

Recently, we have shown that exposure of CPZ to 266 nm laser radiation yields modifications of the absoprtion spectra that are indicative of structural change(s) of the molecule. Of particular interest is that the irradiation process yields a product that has greater activity against the Gram-positive *Staphylococcus aureus* than the parental un-irradiated compound [37]. These preliminary studies supported the concept that exposure of a medicinal compound to a given laser beam of high energy could not only modify its structure, but could also yield one or more species that have greater biologic activity with perhaps a lesser degree of toxicity. Moreover, they further suggested that the formed new species might be intermediates created during the synthesis of CPZ but which due to the pyrogenic methodology used, their identity was not noted.

The study to be described exposed varying concentrations of CPZ to 266 nm wavelength laser beam for increasing periods of time, and at each interval the absorption spectra of the samples were recorded. The samples were also subjected to Thin Layer Chromatography (TLC) and evaluated for biological activity against the test organism *Staphylococcus aureus* ATCC 25923. The products contained in each sample were subjected to High Pressure Liquid Chromatography - Diode Array Detection (HPLC-DAD) and HPLC-MS/MS methods that identified each major component of the samples.

## Materials and Methods

### Characteristics of the laser

**Figure 1:**
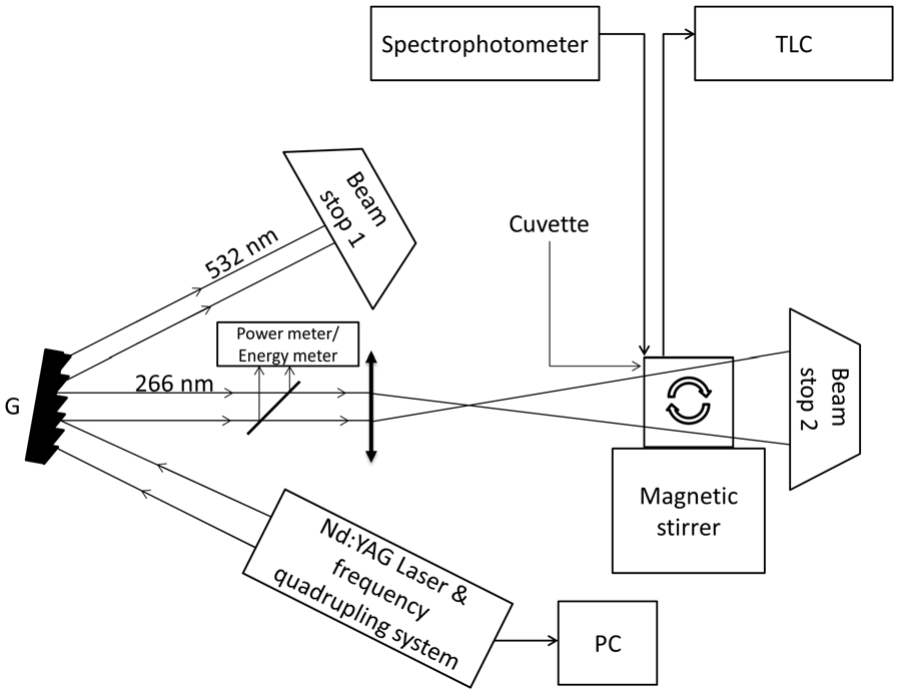
Experimental Set-up. The laser beam incident on the sample is emitted by a Nd:YAG pulsed laser and is crossing an OPO system that allows obtaining a laser beam at 266 nm; the grating G is utilized to separate the second harmonic (532 nm) with respect to the fourth (266 nm) so that on the Cuvette only the 266 nm beam falls. The liquid in the cuvette is agitated by a Magnetic stirrer and the beams that are not useful are blocked by a Beam Stop unit. The laser induced fluorescence emission by the sample is collected with an Optical fiber, and analyzed with a Spectrometer – PC system. The sample exposed/modified to/by laser radiation is characterized by the absorption spectrum measured with a spectrophotometer and by Thin Layer Chromatography (TLC) measurements.

Laser pulses repetition rate: 10 pulses per second; laser beam wavelength, λ = 266 nm, which is the fourth harmonic of the Nd:YAG laser beam emitted at 1.064µm; full time width of the laser pulse: 10 ns; available energy of the laser beam: 6.5 mJ (if not noted otherwise); the laser beam cross section was dimensioned to cover the inner cross section of the cuvette, placed in perpendicular position on the propagation direction of the laser beam. The set-up is described by Figure 1.

### Phenothiazine studied for effects of a 266 nm laser beam

**Figure 2:**
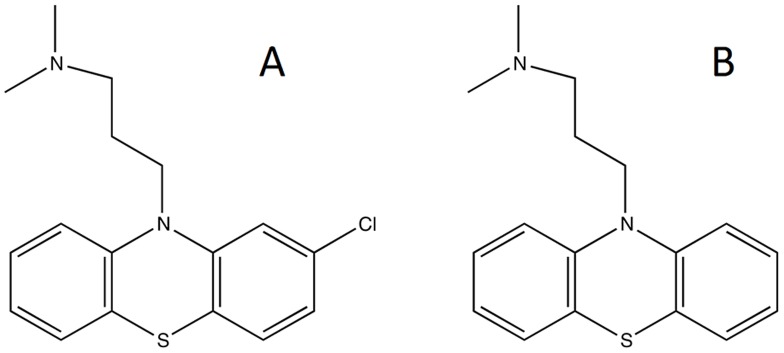
Structures of CPZ (A) and PZ (B).

Chlorpromazine hydrochloride (CPZ) and Promazine hydrochloride (PZ) in powder form were purchased from Sigma, Madrid, Spain. The purity of the compounds was over 98.9%. CPZ and PZ were dissolved at a concentration of 20 mg/mL in distilled water immediately before use and the vials containing the solutions protected from light. The chemical structures of CPZ and PZ are shown in Figure 2.

### Determination of polymerization of a saturated solution of CPZ

In order to insure that the irradiation of CPZ takes place at initial concentrations that do not lead to polymers formation, a polymerization test was performed as in [37] mentioning that in all the experiments no additives were added and only distilled water was used as a solvent. Concentrations as high as 20 mg/mL were shown to express a behavior that will not affect the absorbance spectrum of the control. The temperature at which the measurements were made was 25 °C. The pH of the solutions were measured using a pH-meter (Schott Instruments, model Lab860), and plots were made for more concentrations, irradiation energies and exposure times (curves not shown); for CPZ at 20 mg/mL the measured pH of the un-irradiated solution was 5.6 and it decreased with the exposure time (laser beam energy 6.5 mJ), so that after 4 hrs the pH value was 2.3, which remained constant afterwards. The pH data are consistent with those reported in [38]. The drug molecules like CPZ behave like surfactants and self-associate in water above a critical micelle concentration (*cmc*) [36]. But this concentration is lower with one or more orders of magnitude than the concentration at which polymerizations takes place [39], so that we may consider that CPZ monomers absorb, in the initial solution, the laser radiation. This is in agreement with the proposal made in [40] that self-association of phenothiazines, (in particular CPZ), is a sequential process of adding monomeric units as concentration increases, rather than micellisation according to the pseudophase separation model. On the other hand, the CPZ molar concentration used in our experiments was around 5.6×10^−2^ M (20 mg/mL) and the literature reports [40], [41] display a large range of *cmc* values: 2.2×10^−2^ M to 0.05×10^−3^ M (measured at pH 7.0); the same broad display of values is shown for PZ which at 20 mg/mL has molar concentration of around 6.2×10^−2^ M and the *cmc* according to [41] is 3.6×10^−2^ M. More, surface tension measurements made on CPZ show a value for *cmc* around 10 mg/ml [41]. Absorption spectra measurements were made on samples obtained by dilution of the 20 mg/mL irradiated samples down to the needed concentrations. Given the above mentioned data we may consider that at 20 mg/mL concentration in distilled water, the CPZ absorption at 266 nm is dominated by monomers, although a micelle production process in the first steps may be present. This may be evidenced by the fact that during the exposure, green precipitates are produced in the solution and the samples were mixed up with a magnetic stirrer (VELP Scientifica, AREC.X) at 700 rpm so that they always kept the liquid characteristics but with the color changed in brown (see also Fig. 1).

### Implementation of the irradiation

A 4 mL quartz cuvette containing 1.5 mL of CPZ concentrations ranging from 2 to 20 mg/mL was exposed in the dark to the 266 nm laser beam at varying energy levels (1, 3 and 6.5 mJ) for varying periods of time ranging from 1 minute to 24 hrs. The use of different concentrations and several exposure times was checked in order to identify the optimal exposure conditions related to the optimal bactericide effects of the CPZ.

The cuvette was fitted with a magnetic bar and the laser beam was focused above the magnet so that no interference between the magnet and the laser beam takes place. The cuvette was placed on a magnetic stirrer and the control set to yield approximately 700 revolutions per minute; consequently, continuous mixing was achieved as evident from the introduction of a dye at the bottom of a filled cuvette when within 2 seconds, complete distribution of the dye in the cuvette took place. Care was taken to insure that mixing did not result in the formation of eddies or generation of bubbles. At the end of each interval an aliquot of the contents of the cuvette was diluted to 0.2 mg/mL and the absorption spectrum from 200 to 1500 nm using a Perkin Elmer Lambda 950 UV/VIS/NIR spectrophotometer was determined. Un-irradiated CPZ diluted to 0.2 mg/mL was similarly evaluated and served as the control. For experiments involving the irradiation of PZ, conditions used for irradiation of CPZ and evaluation of irradiation products by absorption spectra and TLC were similarly employed for irradiated PZ and its un-irradiated control.

### Thin layer chromatography (TLC)

1 µL aliquots were taken from the irradiated samples and applied to a normal phase Thin Layer Chromatographic (TLC) silica plate (DC-Alufolien Kieselgel 60F_254_; Merck, Darmstadt, Germany). The mobile phase solvent system consisted of acetone–methanol–ammonia (150∶50∶5, v/v/v). Plates were examined under UV light at 254 and 366 nm and photographed. At 254 nm, the fluorochrome present in the silica plate fluoresces green. Compounds that do not fluoresce will appear as dark spots. At 366 nm, compounds that fluoresce will appear bluish white whereas the background will be dark blue. It is to note that after TLC evaluation, the samples were adjusted to a concentration of 2 mg/mL and TLC was repeated. This provided the opportunity to compare changes produced by irradiation. Permutations of TLC that afforded direct comparison for each interval at each concentration of irradiated CPZ were also performed.

### High Pressure Liquid Chromatograpy - Diode Array Detection (HPLC-DAD)

The 20 mg/mL samples irradiated for different times were analyzed by a gradient HPLC system of two Jasco PU-2080 pumps coupled to a Jasco MD-2010 Plus diode-array detector (DAD) controlled by the ChromNAV chromatographic software. Gradient system was 33% aqueous acetonitrile (ACN) smoothly increasing to 40% in 30 min at a flow rate of 1 mL/min; 100% ACN was reached in 1 min while flow was also increased to 2 mL/min. After 3 min of wash, the solvent composition and flow returned back to the original 33% and 1 mL/min, respectively. Both components of the mobile phase contained 0.05% of trifluoroacetic acid (TFA). An Agilent Zorbax SB-CN (5 µm, 4.6×250 mm; Agilent Technologies, USA) column was used, and 33.0 KB/min of DAD data were collected from 200 to 650 nm at default settings.

### Quantitative determination of CPZ and PZ in the irradiated samples by HPLC-DAD

The 20 mg/mL samples were diluted in duplicates to 2 mg/mL and 10 µL of each was injected. CPZ was determined at 254.7 nm, while for PZ, 250.4 nm was used. Integration of peaks for both compounds were automatically performed at a slope sensitivity of 100.00 µV/s. Detection selectivity for CPZ and PZ was checked by systematically comparing UV spectra between 220 and 300 nm through the corresponding peak. For the calibration curves, stock solutions of 20 and 1 mg/mL for CPZ, and of 1 mg/mL for PZ were prepared and diluted with distilled water as appropriate to obtain 0.005, 0.01, 0.025, 0.05, 0.075, 0.25, 0.75, 1.00, 2.00, 3.00 and 4.00 mg/mL standards for CPZ, and 0.0075, 0.01, 0.025, 0.05, 0.075, 0.1 and 0.2 mg/mL standards for PZ. Above 1 mg/mL concentration linearity decreased for CPZ, hence only standard solutions from 0.005 to 1 mg/mL were used for the calibration curve, resulting in an r^2^ of 0.9996. For PZ calibration, r^2^ was 0.9987.

### High Pressure Liquid Chromatography - tandem Mass Spectroscopy (HPLC-MS/MS)

The 20 mg/mL solution of CPZ irradiated for 24 h was analyzed by HPLC-MS/MS, using a Shimadzu HPLC system consisting of two LC 20AD HPLC pumps, a SIL-20A HT autosampler coupled to a Shimadzu SPD 20A UV/VIS detector and an API 2000 triple quadrupole tandem mass spectrometer (AB SCIEX, Foster City, CA, USA). HPLC conditions were the same as for HPLC-DAD. For MS/MS, electrospray ionization (ESI) was used in the positive mode with the following settings: declustering potential  =  80, focusing potential  =  400, entrance potential  =  10 and T = 300°C. Q1 monitoring (taking single mass spectra) between 50 and 1500 Da, and multiple reactions monitoring (MRM) was performed. Q1 is the first quadrupole of the MS/MS; the MS/MS spectrometer was used as a single mass spectrometer, without fully utilizing the system's features for monitoring selected fragment ions.

### Antimicrobial properties of the irradiated samples

The antimicrobial properties of the parental compounds and the irradiated products were evaluated against *Staphylococcus aureus* ATCC 25923. The strain grown in Mueller-Hinton broth over-night was swabbed onto a Mueller-Hinton agar-containing plate. Filter paper was impregnated with multiple 5 µL aliquots of the control (un-irradiated) and 266 nm laser irradiated products from varying intervals of irradiation, placed on top of the agar containing the swabbed bacteria. The plates were incubated over-night and were examined for presence of clear zones (zones of inhibition) around the disks containing the control un-irradiated CPZ and the products of irradiation. Making the assumption that the “total amount of CPZ” regardless of its alteration by the laser 266 nm beam is present in the sample, amounts of “CPZ sample” (irradiated for varying periods of time), that ranged from 100 to 600 µg in 5 µL were applied to disks. The disks were placed onto the agar plate containing the swabbed bacteria, the plates incubated overnight at 37°C and the zones of inhibition, if present, measured in mm. The MIC of CPZ and irradiated CPZ samples were conducted by the broth dilution method [37]. It is to mention that the solutions exposed to laser radiation contain not only the parent compound (if such a compound or at least a fraction of it remain in the solution) but also the long lived photoproducts which last in the solution. The short lived photoproducts are not acting on the bacteria, since the application of the solution on the bacteria is made at hours or even longer time intervals after ending the irradiation.

## Results

### Evaluation of the products from the irradiation of varying concentrations of CPZ for extended periods of time by the 266 nm laser beam of varying energy levels

**Figure 3:**
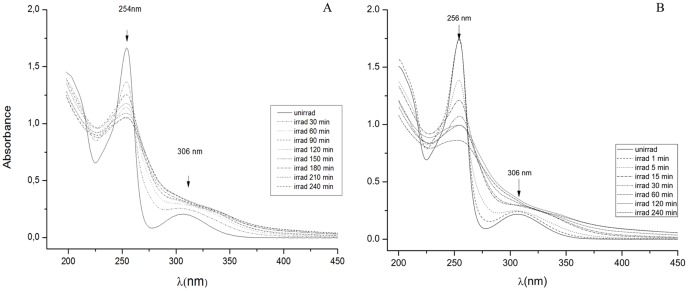
Absorption spectra of the irradiated products per unit period of time. Irradiation of 2 mg/mL of CPZ for intervals of A) 30 minutes up to 240 minutes at E = 1 mJ and B) Irradiation of 2 mg/mL of CPZ from 1 to 240 minutes at E = 6.5 mJ. Note: After irradiation, an aliquot of the contents of the cuvette was diluted to 0.2 mg/mL and the shown absorption spectra were measured. Due to the fact that the absorption peaks are quite broad and the experimental errors to assign their wavelength are associated with the peaks spectral widths, the peak absorbance of control, un-irradiated CPZ varied from 254 to 256 nm.

The conditions employed for standardization of the process for irradiation of a compound must accommodate varying concentrations of the compound exposed to the laser beam; must accommodate a varying period of time of irradiation; and, must accommodate varying energy levels of the laser beam. These conditions must be defined if the irradiation method is to be standardized and the results obtained are to be precise. In addition, if the identity of the products formed from irradiation by laser is to be determined, the amount of sample needed subsequent for chemical analyses must be sufficient. The high sensitivity of HPLC-MS/MS provides a useful tool for screening for expected products, while a complete structure elucidation of the isolated species by NMR needs a larger sample size. Arbitrarily, a concentration of 2 mg/mL of CPZ was selected for initial exposure to a 266 nm laser of varying energy levels for periods of 1 to 240 minutes. As shown by Figure 3A, within 30 min of irradiation of 2 mg/mL of CPZ at an energy level of 1 mJ, the peak absorbance (254 nm) of the product of irradiation is reduced by 17% over that of the un-irradiated CPZ control. By the end of 240 minutes the peak absorbance is reduced by 48%. Concomitant with the reduction of peak absorbance at 254 nm, the second peak of absorbance at 306 nm prominently present in the un-irradiated control, begins to lose its identity by the end of 30 minutes of irradiation, and is completely merged as a sloping shoulder by the end of 240 minutes.

Because the first peak at 254 nm is broad we may consider [42] that it contains contributions of the reaction products obtained by irradiation since the absorption spectrum of PZ irradiated with 266 nm in the same conditions shows a dominating absorption peak centered on 254 nm (data not shown). Increasing the energy of the 266 nm beam to 3 mJ reduces the peak absorbance 254–256 nm at the end of 240 minutes to the same level as that produced by 1 mJ, 52% vs 48%, respectively; the shoulder that masks the 306 nm peak of CPZ is essentially the same as that produced by 1 mJ of energy (data not shown). Increasing the energy of the 266 nm laser to 6.5 mJ produces the spectra depicted by Figure 3B. Briefly, within 5 minutes of irradiation, the peak absorbance 254–256 nm is reduced by 27% and the shoulder that masks the 306 nm second absorbance peak is considerably larger in area than that produced by the 3 mJ energy level. TLC examination of the products of irradiation at 1 and 6.5 mJ for varying periods of time (data not shown) indicated a decrease in the amount of CPZ, which by the end of 240 min of irradiation is barely detectable. In contrast, when the energy level of the laser is increased to 6.5 mJ, CPZ is totally absent by the end of 30 min. This decrease in amount of CPZ is accompanied by the production of more polar species, which reached maximum intensity by the time when all parent CPZ has disappeared (240 min). In contrast, at the irradiation energy level of 6.5 mJ those products are significantly present within 1 minute, and continue to increase until reaching a maximum at the time when all of the un-altered CPZ has disappeared (30 minutes). Moreover, the changes produced by irradiation of the 2 mg/mL of CPZ are more rapid and intense with higher level of irradiation energy. In addition, we may conclude that the majority of the products resulting from irradiation of CPZ by the 266 nm laser beam are very polar compound(s) and remain at the point of application even under the strong solvent conditions used. The CPZ solution at the concentration of 2 mg/mL does not show evidence of aggregate formation (data not shown) and does not fluoresce under a 366 nm lamp, whereas the products of irradiation at low and high energy do fluoresce [43]. We may conclude that irradiation by the 266 nm most likely promotes aggregation, most noticeable at the point of application of the TLC, that present with characteristics of “J” aggregates, namely, fluorescence [44]. However, although “J” aggregates are usually studied in dyes, within a matter of minutes, exposure of CPZ to the 266 nm laser beam, even at the lowest energy level of 1 mJ, yields a reddish brown color that is present at all concentrations of CPZ exposed to white and UV light as found by others [45].

**Figure 4:**
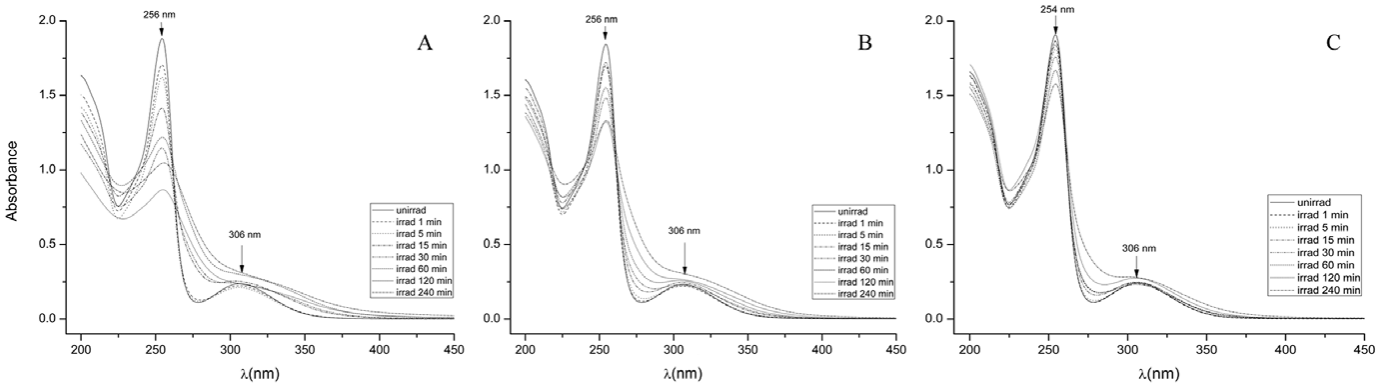
Absorption spectra of irradiated CPZ at concentrations of A) 5 mg/mL, B) 10 mg/mL and C) 20 mg/mL during 240 minutes. E = 6.5 mJ. Note: After irradiation, an aliquot of the contents of the cuvette was diluted to 0.2 mg/mL and the shown absorption spectra were measured.

**Figure 5:**
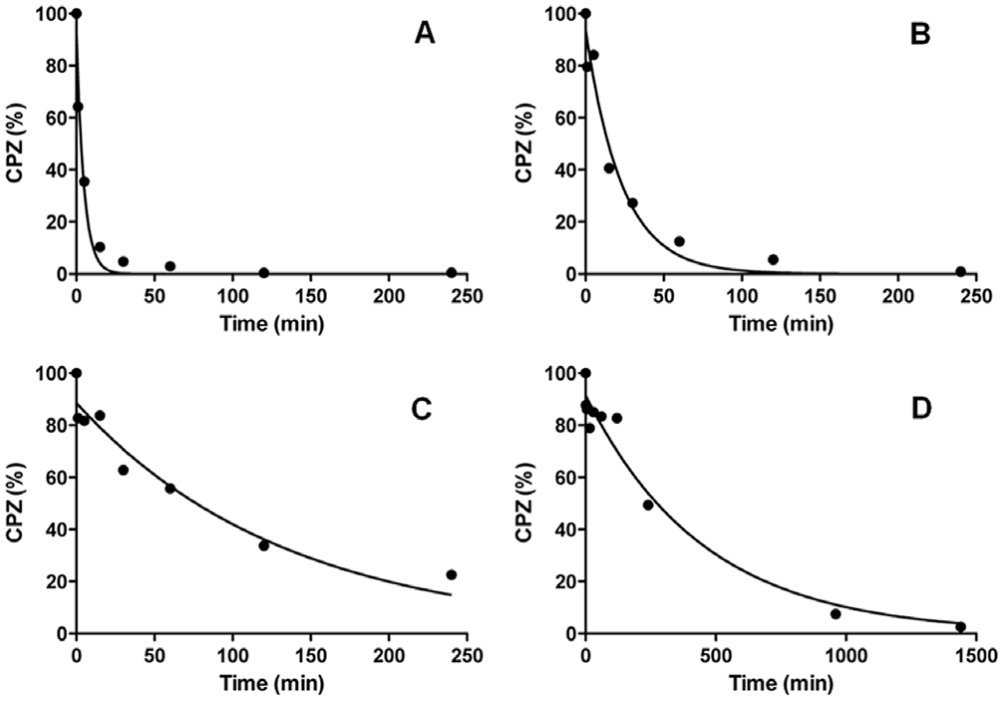
Dynamics of CPZ photolysis at a 2 (A), 5 (B), 10 (C) and 20 (D) mg/mL starting concentration expressed as percentage present in the sample compared with the 100% initial amount. Kinetic curves were fitted according to the one-phase decay exponential model of GraphPad Prism 5.0.

The irradiation of 2 mg/mL of CPZ with the 266 nm laser beam at 6.5 mJ rapidly converts almost all of the CPZ into a product that may in turn interact with the same beam, and this second product may in this manner yield a third, *etc.* According to our observations, increasing the concentration of CPZ to be irradiated under the same conditions as above provides a greater amount of products from irradiation. A higher CPZ concentration in distilled water leads to generation of more photoreaction products in higher amounts. This is the more so if the exposure time is increased. The photoreaction products mentioned below which are identified in the exposed solutions show an increase of quantity evidenced by TLC measurements (data not shown). Once the photoproducts are generated, some of them absorb (such as PZ for instance) or may absorb the 266 nm photons so that, at this stage it is difficult to appreciate which is the amount of light absorbed not only by the parent compound but by the photoproducts as well. In this paper only an overall result of the absorption process is shown. The products from irradiation of higher concentrations of CPZ (5, 10 and 20 mg/mL) by the 266 nm laser beam for varying periods of time (1 minute to 4 hrs) were evaluated by similar absorption spectra and TLC as those conducted for the 2 mg/mL of CPZ. As evident from Figures 4A, B and C, although with time, the intensity of the absorption peak 254–256 nm decreases with prolongation of irradiation for each of the concentrations of CPZ, the difference of intensity between the first peak of the irradiated CPZ and that of the un-irradiated control decreases with concentration of CPZ irradiated. Irradiation of concentrations of CPZ as high as 20 mg/mL, for as long as 240 minutes does not alter the wavelength of the first absorption maximum. In contrast, as the irradiation of increasing CPZ concentrations is prolonged, the formation of the shoulder that begins to fuse with the second absorbance peak 254–256 nm noted with the irradiated 2 mg/mL sample (Figure 4A, B and C), becomes more pronounced as the concentration of exposed CPZ is increased. We interpret these findings to suggest that: **a)** as the concentration of irradiated CPZ is increased, the amount of CPZ that is not altered masks the amount of CPZ that has been altered by the irradiation process, and, **b)** the fusion of the second peak of absorbance at 306 nm with the shoulder that increases with irradiation time is due to the new species formed from irradiation, which is in agreement with [34], [42]. That these interpretations are reasonable is supported by the examination of the products of exposure at increasing concentrations of CPZ to the 266 nm laser beam. Firstly, with irradiation time, it is observed that at all concentrations of exposed CPZ, the amount of CPZ decreases relative to the un-irradiated control (Figure 5). However, as the concentration of CPZ is increased, the amount of CPZ which is not modified is as expected, increased.

**Figure 6:**
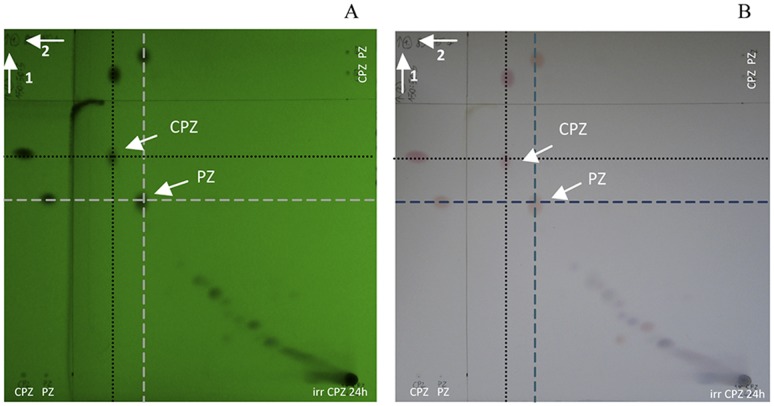
2D-TLC for 20 mg/mL of CPZ irradiated 16 hrs at 266 nm. E = 6.5 mJ. A) visualization under UV light 254 nm; B) TLC sprayed with concentrated H2S04-EtOH-H2O (1∶6∶1, v/v/v)

**Figure 7:**
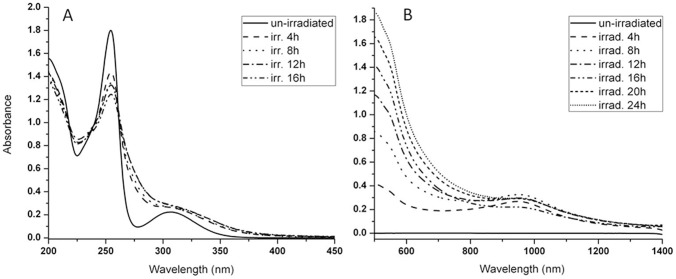
Absorption spectrum of 20 mg/mL of CPZ solution exposed to laser beam; beam energy at 266 nm is 6.5 mJ. A). Irradiation time up to 16 hrs; scanned from 200 to 500 nm. Sample diluted to 0.2 mg/mL after exposure. B). Irradiation time up to 24 hrs; scanned from 500 nm to 1500 nm 20 mg/mL.

In order to optimize the amount of converted products and duration of irradiation, 20 mg/mL of CPZ was irradiated under the same conditions for periods of time up to 24 hrs. The 16 hrs exposure, results in the reduction of the absorbance peak 254–256 nm (data not shown) much like that evident after the irradiation of the 2 mg/mL CPZ for 4 hrs (see Figure 3A). TLC analysis showed a prominent band behind the CPZ, which was identified as PZ by 2D-TLC. As noted under examination by the 254 nm UV lamp and depicted by Figure 6A, the prominent band behind CPZ is PZ. Lastly, as noted by Figure 6B, whereas the prominent PZ band seen in the TLC under the 366 nm does not fluoresce, the species of the irradiated sample that migrate behind the prominent band fluoresce as does the very polar material that remained at the point of application. As noted by Figure 7A, after 16 hrs of irradiation of 20 mg/mL of CPZ, the intensity of the peak absorbance 254–256 nm is reduced to about 60% of the un-irradiated CPZ control and the formed shoulder masks the underlying 306 nm second peak of absorbance. After further irradiation of up to 24 hrs, the peak absorbance associated with CPZ at 254–256 nm remains at the level noted for 16 hrs of irradiation. Because irradiation of 2 mg/mL of PZ for 4 or more hours does not reduce the peak absorbance at 254–256 nm by more than 50% (data not shown), the maximum reduction of the peak absorbance at 254–256 nm noted at the irradiation of CPZ after 16 and 24 hrs probably accounts for the presence of PZ. This is due to the fact that almost all of the CPZ has disappeared as noted by TLC evaluation of the 16 and 24 hrs CPZ irradiated samples. Irradiation of PZ, as is the case with CPZ, does not yield products that are more polar than PZ and these fluoresce under the 336 nm UV lamp (data not shown). These products show similarities to those that are produced by the irradiation of CPZ suggesting that the interaction of serially produced new species increases with time, as would be expected. In addition, the absorption spectrum from 500 to 1500 nm of CPZ under prolonged irradiation yields a peak absorbance of *ca.* 500 nm and a second peak at *ca.* 950 nm both of which increase with duration of irradiation (Figure 7B). No absorbance by un-irradiated CPZ is noted between 500 and 1500 nm. Because NIR and IR spectra of derivatives of CPZ suggest the presence of polymers, these results suggest that with duration of irradiation, the formation of polymers probably takes place as predicted by others [34], [46], [47].

### Analysis of the products of irradiation of 20 mg/mL of CPZ by HPLC-DAD and HPLC-MS/MS

A variety of HPLC columns and mobile phases of different selectivity were tested for optimizing separation conditions. Over a Zorbax SB-C18 and an Eclipse XDB-C8, the SB-CN column with a TFA-containing mobile phase showed the best performance for separating the photodegradation products of CPZ. Moreover, due to the ability of cyano phase for less bonding strong bases, this column was the less sensitive for nearly irreversibly retaining, possibly polymers.

**Figure 8:**
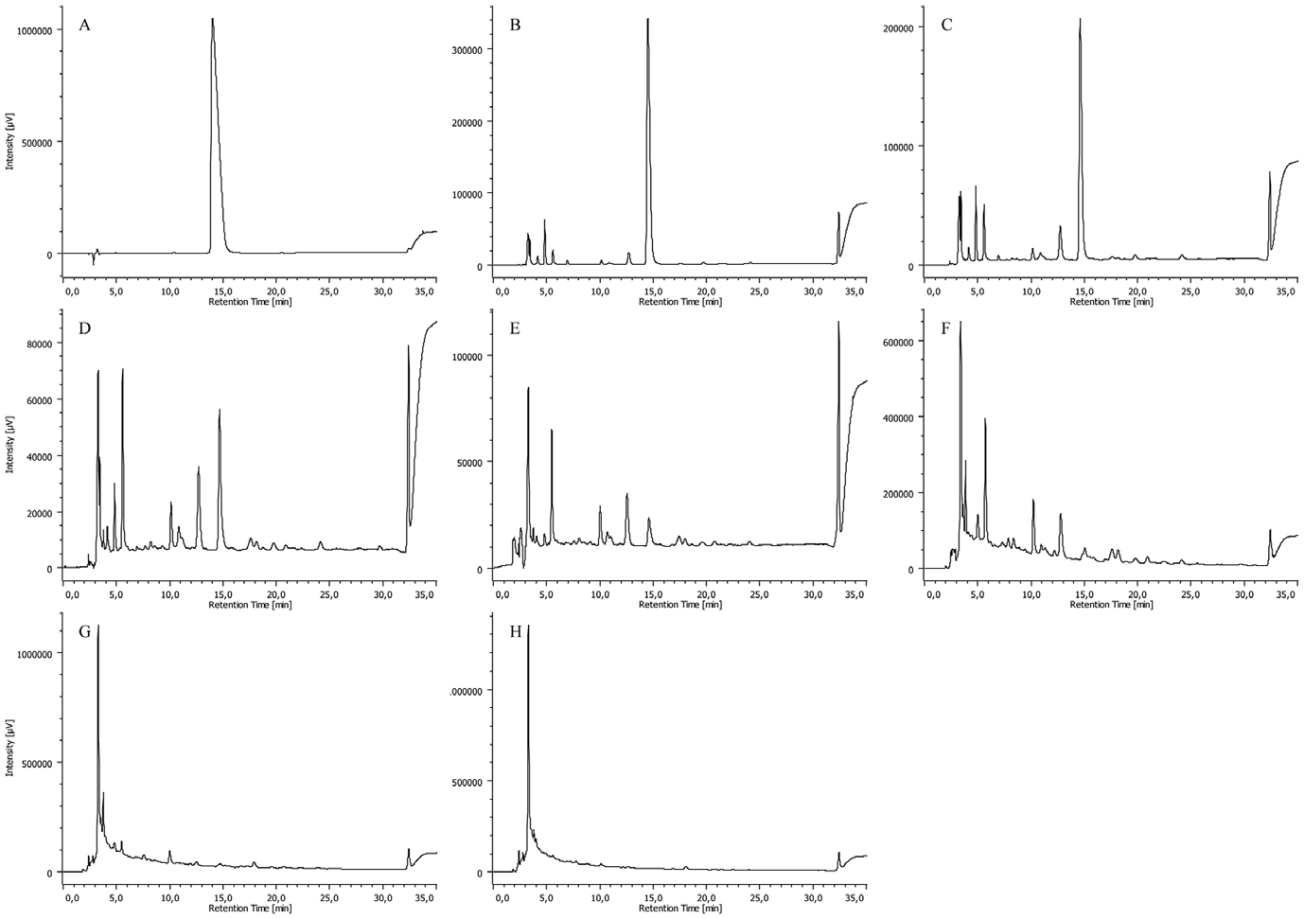
HPLC-DAD chromatograms of un-irradiated 2 mg/mL of CPZ (A) and 2 mg/mL of CPZ irradiated for time periods of 1 (B), 5 (C), 15 (D), 30 (E), 60 (F), 120 (G) and 240 (H) minutes, recorded at the maximum absorbance in the wavelength range of λ = 230–450 nm. Note: intensity scales are not identical; overall amount of UV-active material is gradually decreasing with the irradiation time.

**Figure 9:**
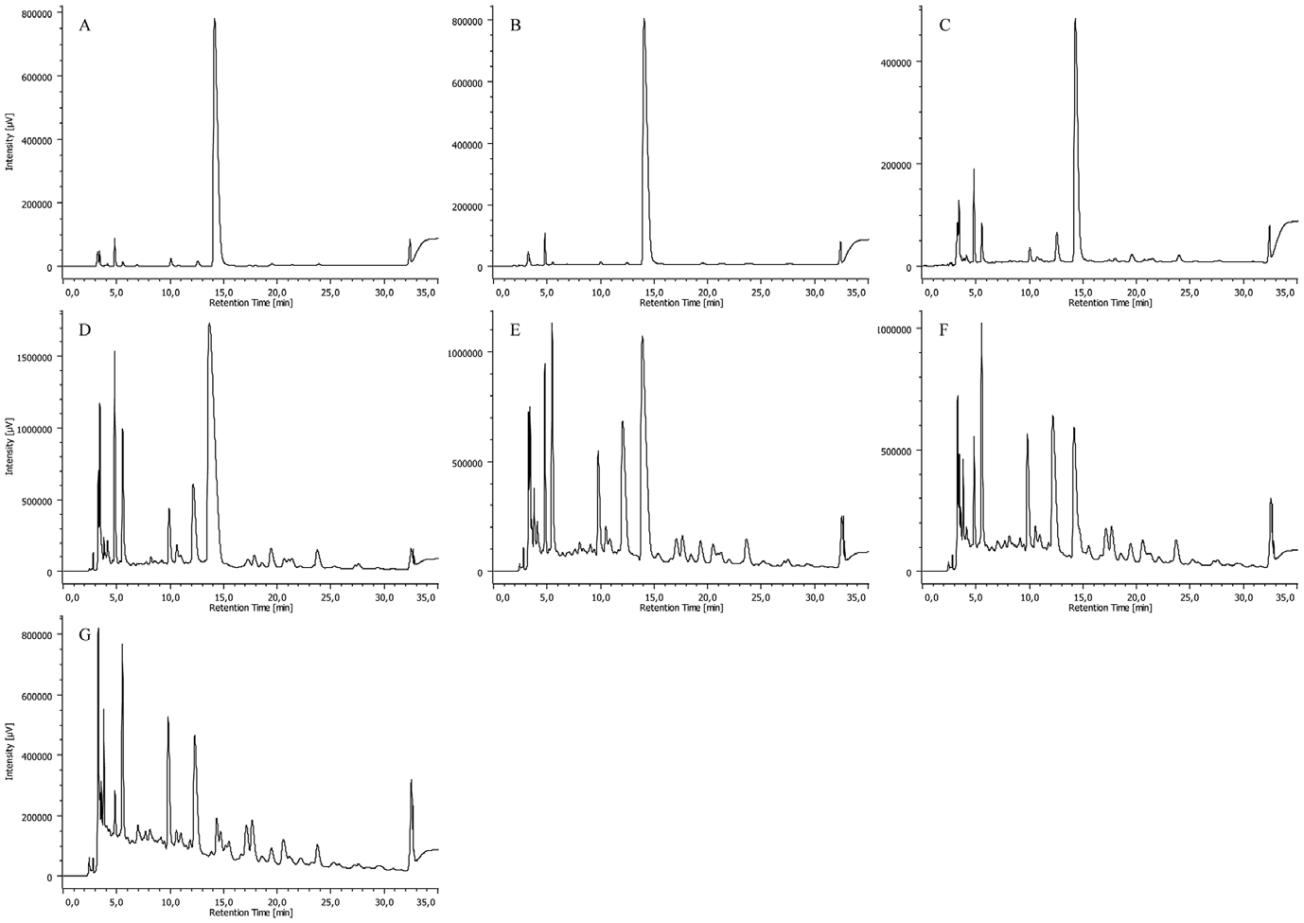
HPLC-DAD chromatograms of 5 mg/mL of CPZ irradiated for time periods of 1 (A), 5 (B), 15 (C), 30 (D), 60 (E), 120 (F) and 240 (G) minutes, recorded at the maximum absorbance in the wavelength range of λ = 230–450 nm. Note: intensity scales are not identical; overall amount of UV-active material is gradually decreasing with the irradiation time.

**Figure 10:**
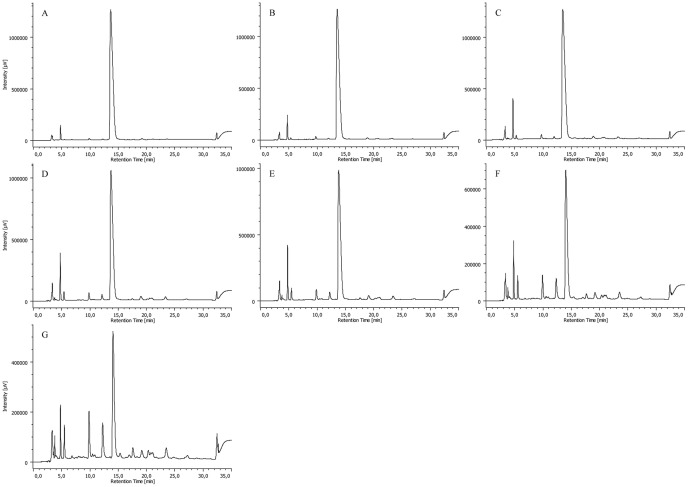
HPLC-DAD chromatograms of 10 mg/mL of CPZ irradiated for time periods of 1 (A), 5 (B), 15 (C), 30 (D), 60 (E), 120 (F) and 240 (G) minutes, recorded at the maximum absorbance in the wavelength range of λ = 230–450 nm. Note: intensity scales are not identical; overall amount of UV-active material is gradually decreasing with the irradiation time.

**Figure 11:**
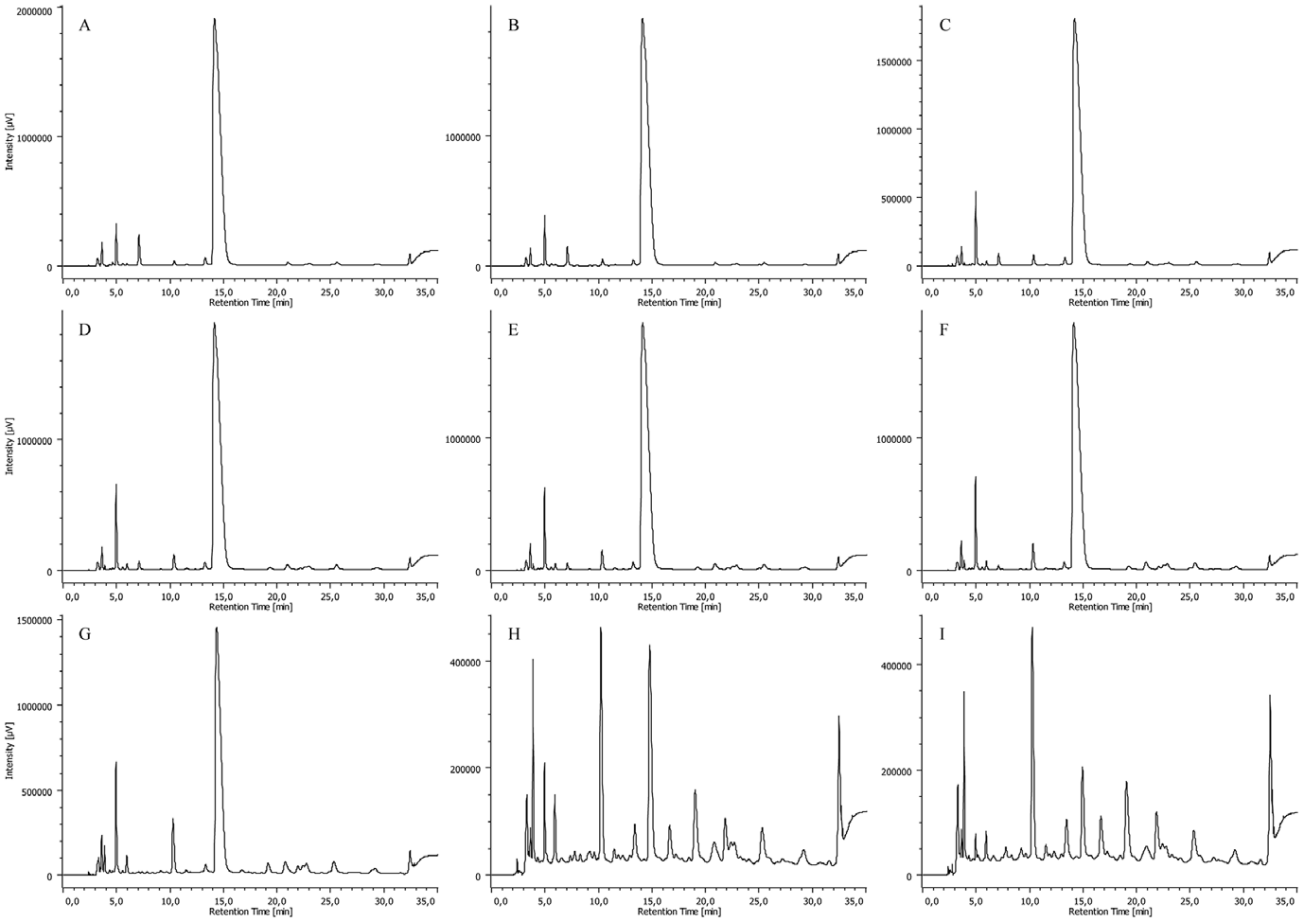
HPLC-DAD chromatograms of 20 mg/mL of CPZ irradiated for time periods of 1 (A), 5 (B), 15 (C), 30 (D), 60 (E), 120 (F) and 240 (G) minutes, and 16 (H) and 24 (I) hours, recorded at the maximum absorbance in the wavelength range of λ = 230–450 nm. Note: intensity scales are not identical; overall amount of UV-active material is gradually decreasing with the irradiation time.

As expected, a gradual decrease in the amount of CPZ was observed by analyzing the samples obtained after different times of irradiation. Figures 8, 9, 10 and 11 summarize the maximum-absorbance chromatograms obtained after different irradiation times for the different initial concentrations of CPZ.

**Figure 12:**
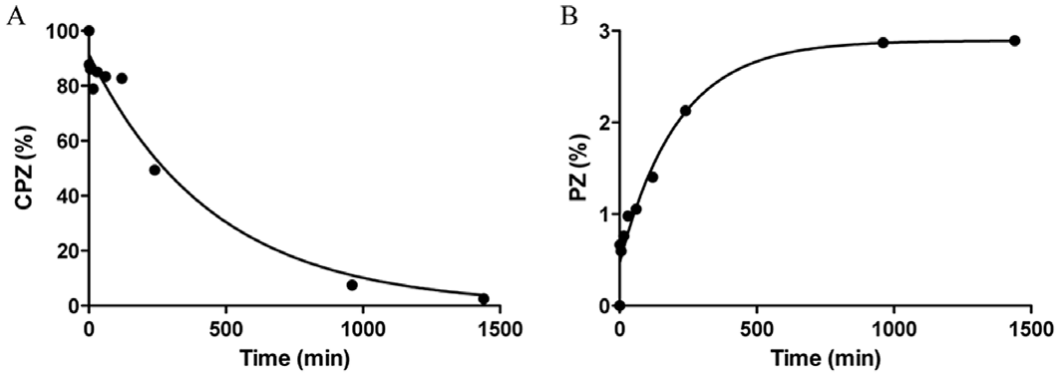
Dynamics of CPZ photolysis at a 20 mg/mL starting concentration (A), accumulating the amount of PZ (B) expressed as percentage present in the sample. Kinetic curves were fitted according to the one-phase decay exponential model of GraphPad Prism 5.0.

Based on our results, PZ was the major compound formed among the UV active products during the irradiation. To investigate the kinetics of the photolytic process, a quantitative determination was performed by HPLC-DAD. The selectivity of detection was investigated for both compounds by checking peak purity based on a systematic comparison of UV spectra along the peak, at wavelengths between 230 and 300 nm. The lowest purity was found for CPZ after 24 hrs of irradiation, when 82.76% of the peak area possessed a purity of over 99%; 14.12% of it was found 95–98.99% pure, and purity of 2% of the peak area was found 90–94.99%, indicating around 3% estimated overall error in the quantification. As for all other samples much better results were obtained; satisfactory detection selectivity and consequentially high determination accuracy for both compounds could be concluded. By using GraphPad Prism 5.0 software, the one-phase decay nonlinear model was found to give the best curve fitting result, when the ‘plateau’ parameter of CPZ was set to zero. The same kinetics fitted well to the PZ/CPZ ratios, showing that the degradation process of CPZ and the formation of PZ are both proportional to the amount of the remaining CPZ. Percentage amounts for these two compounds present after various irradiation times for the 20 mg/mL starting concentration and results of the curve fittings are shown in Figure 12.

The 20 mg/mL sample of CPZ after a 24 hrs irradiation was also analyzed by HPLC-MS/MS. Q1 monitoring of the chromatogram revealed the presence of a very large number of major degradation products, most of which were not recognizable with UV detection. Moreover, when overviewing the total mass spectrum, polymers reported before were hardly detectable.

**Figure 13:**
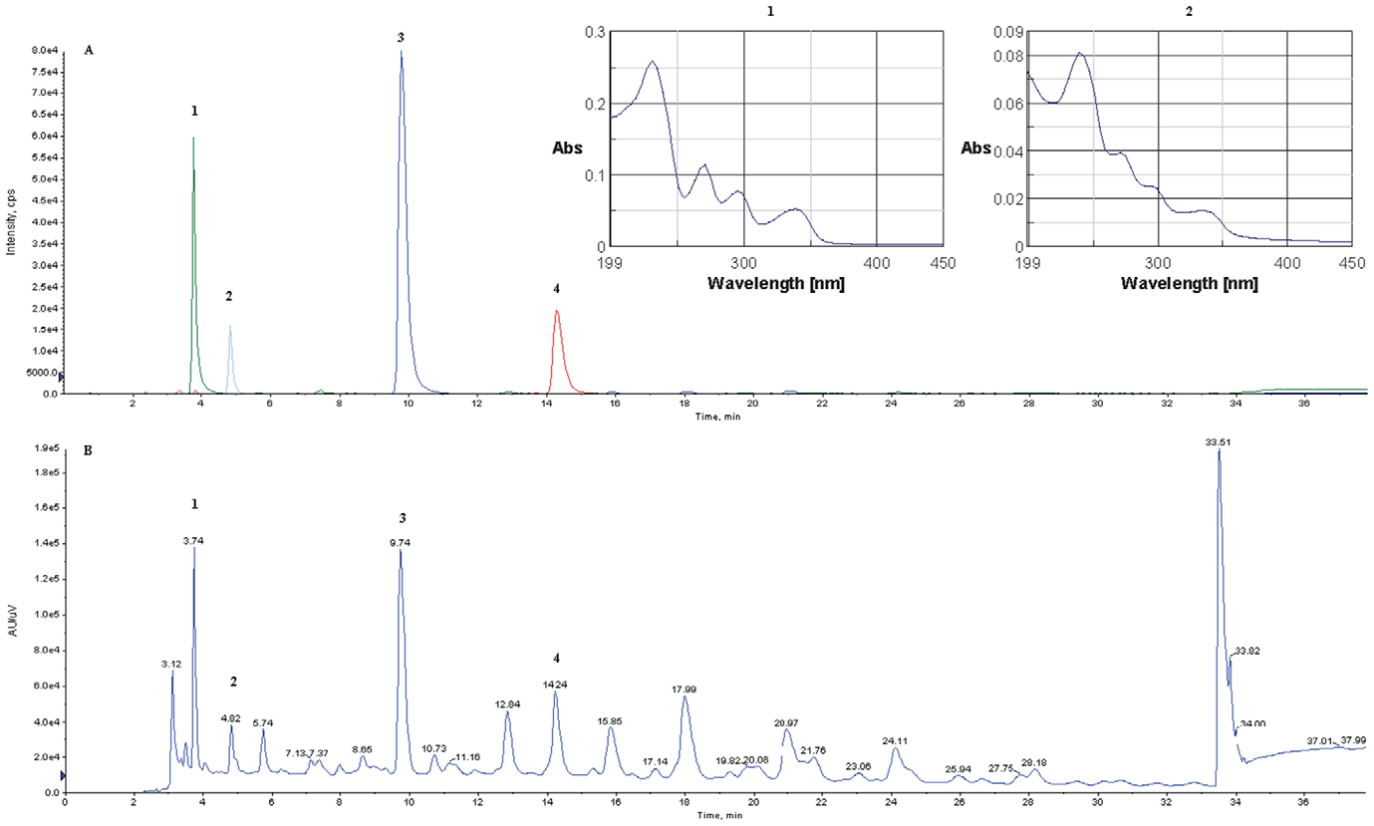
HPLC MS/MS. Multiple reactions monitoring (MRM) was performed for [M+H+]  = 285, 301, 317, 319 and 335, in order to obtain selective chromatograms for PZ (3), hydroxypromazine or PZ sulfoxide (1), hydroxypromazine sulfoxide (2), CPZ (4) and CPZ sulfoxide, respectively (A). Identity of PZ sulfoxide over hydroxypromazine for peak 1 was proven based on the on-peak UV spectrum obtained from the HPLC-DAD chromatogram; the four maxima are characteristic for phenothiazine sulfoxides. UV chromatogram recorded during the MRM experiment at 240 nm is also shown (B).

Multiple Reaction Monitoring (MRM) was used for selective detection of [M+H+]  =  285, 301, 317, 319 and 335 (PZ, hydroxypromazine or PZ sulfoxide, hydroxypromazine sulfoxide, CPZ and CPZ sulfoxide, respectively). As seen from Figure 13, four out of these previously reported degradation products of CPZ could selectively be identified, among which the [M+H+]  = 301 peak could either be hydroxypromazine or PZ sulfoxide. The identity of this peak was solved based on the HPLC-DAD results, where the on-peak UV spectrum at this retention time showed four maxima, characteristic of phenothiazine sulfoxides; hence this peak was identified as PZ sulfoxide.

#### Bioactivity of the products of irradiated CPZ

Our results demonstrated that prolonged irradiation for up to 4 hrs yielded a much more active product on the *Staphylococcus aureus* ATCC 25923 than the control un-irraditated CPZ [37]. A similar approach was taken for the evaluation of activity of products of CPZ subsequent to irradiation by the 266 nm laser beam.

**Figure 14:**
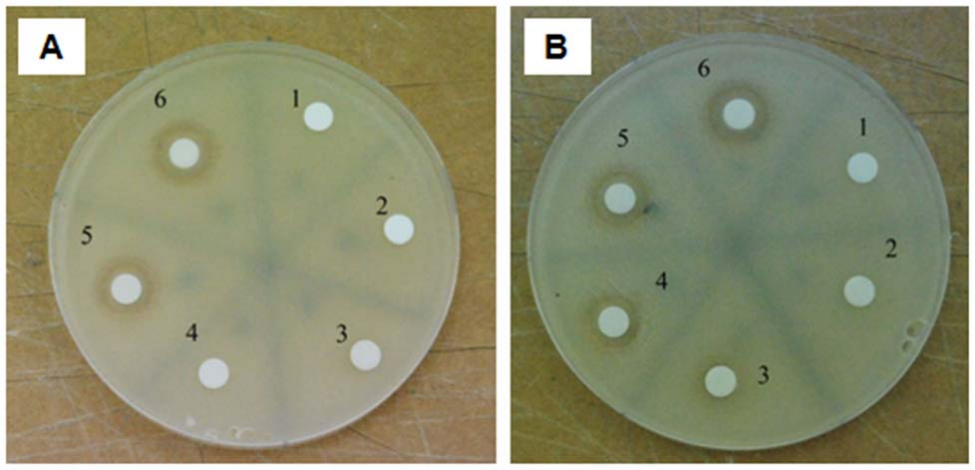
The effect of irradiation of 20 mg/mL of CPZ on the antimicrobial activity of CPZ. (A).CPZ @ 20 mg/mL irradiated for 1 minute. B). CPZ @ 20 mg/mL irradiated for 240 minutes. Amounts of “CPZ” of sample applied to disks: 1 (100 µg); 2 (200 µg); 3 (300 µg); 4 (400 µg); 5 (500 µg); 6 (600 µg). Note that the # 4 disk containing 400 µg of “CPZ” 20 mg/mL sample irradiated 240 minutes produces a zone of inhibition whereas the disk that contains the same amount of “CPZ” 20 mg/mL irradiated 1 minute does not produce a zone of inhibition. These results suggest that the antimicrobial activity of the CPZ exposed to a 266 nm laser beam for 240 min. is increased.

As noted by Figure 14, whereas 20 mg/mL of CPZ irradiated for 1 min produced a zone of inhibition with 500 µg applied to the disk, prolonged irradiation for 240 min produced zones of the same diameter with a 300 µg application of the product of irradiation. Irradiation of 20 mg/mL of CPZ for 24 hrs was evaluated for its MIC against *Staphylococcus aureus* ATCC 25923 reference strain by the broth dilution method. Whereas the MIC of control non-irradiated CPZ was 60 µg/mL, that of the 24 hrs irradiated CPZ was 3.75 µg/mL. These results confirmed previous observations that indeed, irradiation yielded a product that was far more (8 fold) active against the test organism than CPZ [24]. Nevertheless, as is the case with CPZ, activity of the 24 hrs irradiated sample is bacteriostatic at the concentrations employed (maximum of 20 mg/mL).

## Discussion

Because therapy of the psychotic patient with CPZ produces phototoxicity in some patients [31], many studies were conducted to determine whether this effect was due to light, specifically UV [30]. These studies showed that white light [28], incoherent UV light [31], coherent UV [48] and “flash photolysis” by a 266 nm laser [32], produced changes in structure of CPZ. The changes were perceived from indirect measurements that suggested the formation of radicals leading to the substitution at the sulfur atom (sulfoxides) and loss of the chloride at C-2 forming PZ. These changes were also perceived as “destruction” of the CPZ molecule, and the use of light to bring about this “destruction” was termed “photolysis” [27], [34], [35], [49]. Nevertheless, because the energy of a laser beam is thousands of times greater than that generated by UV light sources, we considered the possibility that lasers could be used to rapidly modify molecules with prolongation of exposure to hours rather than nanoseconds [32] and perhaps be used to produce reactions not possible with routine pyrogenic techniques used in organic chemistry. The CPZ is the second most studied compound next to aspirin (search in PubMed yielded 20,000 articles on CPZ versus 49,000 for aspirin). Much information on the response of CPZ to light has been generated for over 50 years [21]–[23], [25]–[27], [29], [30], [33]–[35], and starting from this we began a series of studies that evaluate the effect of a high energy laser beam on the structure of CPZ and correlate these with its bioactivity of against bacteria [37] and cancer.

A particularly interesting study is reported in [29] where are described effects of CPZ and THR modified by exposure to incoherent optical beams on biological targets in environment water. These data are complementary to our reports, and there are important differences between them which are mentioned in the following:

- in [29] the authors approach the subject from the point of view of the photodegradation of the CPZ and THR in environmental water under the interaction with incoherent, continuous wave (cw) light emitted in the visible and UV-A (normally by the sun). This is made in the context of studying the toxicity of the phenothiazines after photodegradation in open environment conditions. Consequently, they expose the samples at two cw light beams which have broad cross sections: one in the visible and the second in the UV-A at 350 nm. In the present paper the approach is given with the purpose to find new ways to fight multiple drug resistance (MDR) developed by bacteria, and we look at the CPZ and THR as medicines that could be modified or could generate photoproducts by exposure to laser radiation, able to have better bactericide effects. With this purpose we expose water solutions of phenothiazines at 266 nm (UV– C) pulsed laser radiation, at energies of the laser beam orders of magnitudes (more than three) higher than in [29].
- Due to the different light sources utilized in the two papers, another difference is that in [29] the solutions were exposed long time intervals (7 days) at visible and 350 nm beams, whereas in our paper the longest exposure time was 24 hours, observing that significant modifications of the CPZ for instance, may be obtained after some hours (2–4 hrs) of exposure to the laser beam.
- Another important difference related to the significantly different irradiating beams is that the maximal concentrations of the phenothiazines in distilled water solutions exposed to optical radiation is in [29] 100 mg/L whereas in our paper 20 g/L. This may lead to different behavior of the photoreaction products obtained following exposure; in [29] almost total destruction of the CPZ is reported after a few hours of exposure at 350 nm. In this paper and in [37] we report almost total destruction of the CPZ after 4 hours of exposure to 266 nm, but at 2 mg/mL concentration. At larger concentrations in our case the parent product is not completely destroyed, and the photoreaction products are stable, i.e. they coexist in the solution months after exposure, so that we are able to make extensive reproducibility experiments. On the other hand in [28] and in this paper it is confirmed that a multitude of photoreaction products are obtained from parent phenothiazines after exposure to optical beams.
- The toxicity of the solutions of CPZ and THR was studied in [29] on biological targets which exist in water, such as protozoan Spirotox (Spirostomum ambiguum) and anostracan crustacean Thamnoscephalus platyurus. In this paper we applied the solutions exposed to laser radiation on Gram - positive bacteria which are usually responsible for infections in humans and animals in the process of wound healing.

Our study required that the method for exposure of a compound to a laser beam be standardized if a laser is to be used for synthesis of new derivatives from a laser sensitive parental compound [37]. Standardization of the laser based method is important because the response of a compound to a specific set of conditions will yield only one chemical reaction. To standardize the method, a number of variables were defined. Firstly, rather than conducting exposure of CPZ dissolved in a variety of solvents to the laser as others have done with UV, we selected distilled water as the first solvent in order to minimize reactions that are solvent dependent. Secondly, we selected a 266 nm laser beam because the peak absorbance of CPZ is at 254–256 nm, and modification of the CPZ was thereby assured. Thirdly, unlike our previous study where the energy level of the laser beam was 8.5 mJ, we empirically selected a 6.5 mJ energy level because it was the lowest energy level that would completely transform a minimum concentration of CPZ within a short period of time (30 minutes).

With the maintenance of the aforementioned conditions, the studies considered concentrations of CPZ in distilled water exposed to the 266 nm laser beam for prolonged periods of time. Under these rigid conditions the sequential development of products from exposure to the 266 nm could be studied. Briefly, with prolonged exposure to the laser beam, at any given concentration of CPZ, the amounts of products identified via TLC increased. With no exceptions, all of the products formed were more polar than the control un-irradiated CPZ. Irradiation of the smallest concentration of CPZ (2 mg/mL) resulted in the complete conversion of CPZ into products that fluoresced under the 366 nm UV lamp whereas CPZ did not emit fluorescence. Increasing the concentration of CPZ exposed to the laser afforded the identification of TLC products that could not be detected with the smallest concentration of CPZ (2 mg/mL). Prolongation of exposure increased the presence of these products such that by the end of 16 hrs, the major product was one that migrated behind CPZ in the TLC system. This product could be seen to decrease with further prolongation of exposure to 24 hrs and was identified as PZ. Careful examination of the 4 hrs TLC of the 2 mg/mL of CPZ shows that a product that migrates closely behind CPZ begins to appear concomitantly with the transformation (disappearance) of CPZ. Interestingly, this product emits fluorescence and is not seen with exposures of higher concentrations of CPZ, nor is it observed with prolonged exposures of concentrations higher than 2 mg/mL. We interpret these findings to suggest that the rapid formation of products from CPZ at its lowest concentration yields a radical that is short lived and slightly more polar than CPZ and that it may represent a species that contains the sulfoxide previously suggested from flash photolysis of CPZ [32]. Monitoring the pH of the 2 mg/mL of CPZ during exposure indicated a rapid drop of 3 to 4 pH units within 5 minutes that was maintained for the duration of the two hour exposure (data not shown). This suggests that the protons were generated from the reaction that leads to sulfoxides as observed by others [31]. All our measurements were made in distilled water and aerobic conditions were always maintained for the samples. No additives were added in the solutions and generally we kept the conditions in which the drug is utilized. So, the sulfoxides formed from exposure of CPZ to UV [50], [51], are accompanied by protons having their source in water. Due to the complexity of the formation process of the photoproducts it is difficult to appreciate at this stage of research the sequence of photoproducts formation and the rates of their formation. Most probably the first step in a complex chain of reaction is the formation of PZ. The pH drop suggests that the CPZ-SO formation, associated with the formation of hydrogen ions is produced through the following pathway [52]:

CPZ + hν (@266 nm) → CPZ^+^ + e^-^

CPZ^+^ + H_2_O → CPZ-SO + e- +2H^+^

CPZ-SO + H_2_O → CPZ-SO_2_ +2e^-^ + 2H^+^

The sequence and the rates of formation may be clarified function of the rigorous identification of the photoreaction products. On the other hand, the killing potential of the irradiated solutions increases with the concentration and the irradiation time as shown in Figure 14.

Our previous results indicated that exposure of 20 mg/mL of CPZ to a 266 nm laser yielded a product with greater biological activity against the test organism *Staphylococcus aureus* ATCC 25923 [37]. This increased activity is further noted in the current study, especially with the results obtained with the 24 hrs irradiation of 20 mg/mL of CPZ. Although the mechanism of action accounting for the increased antibacterial activity produced by laser exposure is not yet studied, phenothiazines such as CPZ have been shown to inhibit efflux pumps of bacteria [6] and of significant importance, promote the killing of intracellular mycobacteria [7], [8], [11], [13]. Because phenothiazines that affect the viability of *Staphylococcus aureus* ATCC 25923 in general also affect that of Mtb [53], and a number of important genes are similar in both species [54]–[57], we would anticipate that the irradiated CPZ would also have bioactivity against Mtb. The fractionation, isolation and characterization of products would afford their direct evaluation for bioactivity against CPZ sensitive cells, including multi-drug resistant cancer [58], [59]. Lastly, because PZ has very little activity against bacteria [60], the increased antibacterial activity of the 24 hrs irradiated 20 mg/mL sample must be due to species other than PZ.

The evaluation of the products from exposure to the 266 nm laser beam by HPLC-DAD and HPLC-MS/MS indicated the presence of 4 major produced species. The amounts of these products increased with duration of exposure such that by the end of 24 hrs, the parental compound CPZ is no longer present and PZ, hydroxypromazine or PZ sulfoxide, hydroxypromazine sulfoxide and CPZ sulfoxide account for the major products present. However, at least two hundred other products are present which due to inadequate amount of sample, could not at this time be identified. Moreover, preparatory HPLC methods will afford the isolation of many of these new compounds which can then be subjected for assessment of biological activity. The identification of many, if not all of the products formed from prolonged irradiation with the 266 nm laser beam, may afford their detection in other systems that produce derivatives such as the enzymatic system present in the liver. This system can yield as many as 40 compounds when the patient is treated with a single dose of CPZ. These compounds are not easily noted with pyrogenic systems since they are short lived and the stopping of any pyrogenic based chemistry is difficult. In the case of the laser based method, the process is immediately stopped when the beam is removed. Lastly, it should be noted that the spectrophotometric and TLC properties of the products formed from laser exposure are stable for at least three months. This degree of stability means that other laboratories that pursue the laser based method are assured of obtaining the same products from the irradiation of CPZ by the 266 nm laser radiation, thereby facilitating further studies which together will complete the chemistry involved in the sequential formation of new species by exposure to a laser beam. Application of the method to other bioactive compounds is encouraged.
