# Correction: Exposure of Chlorpromazine to 266 nm Laser Beam Generates New Species with Antibacterial Properties: Contributions to Development of a New Process for Drug Discovery

**DOI:** 10.1371/annotation/1ba396af-ad78-4740-b9f8-93cf03661b35

**Published:** 2013-03-29

**Authors:** Mihail Lucian Pascu, Balazs Danko, Ana Martins, Nikoletta Jedlinszki, Tatiana Alexandru, Viorel Nastasa, Mihai Boni, Andra Militaru, Ionut Relu Andrei, Angela Staicu, Attila Hunyadi, Seamus Fanning, Leonard Amaral

The funding statement was incorrect. The correct funding information is:

This work was supported by a grant of the Romanian National Authority for Scientific Research, CNCS – UEFISCDI, project number PN-II-ID-PCE-2011-3-0922. It was also supported by the New Hungary Development Plan (TÁMOP-4.2.1/B-09/1/KONV-2010-0005), the Baross Gábor Program (MFB-00339/2010) and partially supported by grants EU – FSE / FEDERPOCI / SAU - MMO/59370/2004 and EU – FSE / FEDER-PTDC/BIAMIC/71280/2006 provided by the Fundacao para a Ciencia e a Tecnologia (FCT) of Portugal. L. Amaral was supported by BCC grant SFRH/BCC/51099/2010 provided by the Fundação para a Ciência e a Tecnologia (FCT) of Portugal and PTDC/SAU-FCF/102807/2008. A. Hunyadi was supported by an STSM within the COST Action CM0804 and B. Danko was supported by an STSM within the COST Action BM0701. A. Militaru and V. Nastasa were supported by projects POSDRU 107/1.5/S/80765 and POSDRU/88/1.5/S/56668, respectively. The funders had no role in study design, data collection and analysis, decision to publish, or preparation of the manuscript. 

